# Differential contribution of basic residues to HIV-1 nucleocapsid protein’s nucleic acid chaperone function and retroviral replication

**DOI:** 10.1093/nar/gkt1227

**Published:** 2013-11-28

**Authors:** Hao Wu, Mithun Mitra, M. Nabuan Naufer, Micah J. McCauley, Robert J. Gorelick, Ioulia Rouzina, Karin Musier-Forsyth, Mark C. Williams

**Affiliations:** ^1^Department of Physics, Northeastern University, Boston, MA 02115, USA, ^2^Department of Chemistry and Biochemistry, Center for RNA Biology, and Center for Retrovirus Research, The Ohio State University, Columbus, OH 43210, USA, ^3^AIDS and Cancer Virus Program, Leidos Biomedical Research, Inc., Frederick National Laboratory for Cancer Research, Frederick, MD 21702, USA and ^4^Department of Biochemistry, Molecular Biology, and Biophysics, University of Minnesota, Minneapolis, MN 55455, USA

## Abstract

The human immunodeficiency virus type 1 (HIV-1) nucleocapsid (NC) protein contains 15 basic residues located throughout its 55-amino acid sequence, as well as one aromatic residue in each of its two CCHC-type zinc finger motifs. NC facilitates nucleic acid (NA) rearrangements via its chaperone activity, but the structural basis for this activity and its consequences *in vivo* are not completely understood. Here, we investigate the role played by basic residues in the N-terminal domain, the N-terminal zinc finger and the linker region between the two zinc fingers. We use *in vitro* ensemble and single-molecule DNA stretching experiments to measure the characteristics of wild-type and mutant HIV-1 NC proteins, and correlate these results with cell-based HIV-1 replication assays. All of the cationic residue mutations lead to NA interaction defects, as well as reduced HIV-1 infectivity, and these effects are most pronounced on neutralizing all five N-terminal cationic residues. HIV-1 infectivity in cells is correlated most strongly with NC’s NA annealing capabilities as well as its ability to intercalate the DNA duplex. Although NC’s aromatic residues participate directly in DNA intercalation, our findings suggest that specific basic residues enhance these interactions, resulting in optimal NA chaperone activity.

## INTRODUCTION

The retroviral nucleocapsid (NC) protein is the major nucleic acid (NA) binding domain of the Gag polyprotein, which is known to be necessary for virion assembly, as well as viral genome (gRNA) selection and packaging (1,2). The major domains of Gag include NC, capsid (CA), matrix (MA) and p6. After Gag processing by human immunodeficiency virus type 1 (HIV-1) protease, CA re-assembles to form a mature virus CA, NC binds to the gRNA inside the CA and MA binds to the cellular plasma membrane (3). Although the sequences of NC domains of Gag vary across different retroviruses, they are generally highly cationic in character (4,5). An exception is HTLV-1 NC, which overall is neutral but contains several highly charged regions (6). One or two CCHC-type zinc fingers, each containing one or two aromatic residues, are common structural elements present in all orthoretroviral NC proteins. The aromatic residues appear to confer some of the specificity of gRNA selection by NC (7,8). In contrast, NC’s basic residues are typically distributed over the entire protein, and are generally responsible for nonspecific electrostatic interactions with NAs (9–14).

HIV-1 NC is a NA chaperone protein that facilitates the rearrangement of NAs into their lowest energy configuration (2,15–23). These rearrangements are essential for many viral replication processes, such as reverse transcription and recombination (14,24–28). Three key steps of reverse transcription, tRNA primer annealing (29), minus-strand transfer and plus-strand transfer (30–34), require significant rearrangement of NA secondary structure. Previous work demonstrated that HIV-1 NC greatly facilitates these processes through its chaperone activity, which includes NA aggregation, destabilization and rapid protein–NA interaction kinetics (15,16,18,35–39). *In vitro* studies suggest that the ability of NC proteins to aggregate NA is due to their cationic character, and this activity is largely independent of their specific zinc-finger structures (9,36,40). In contrast, the other major component of the chaperone activity, NA destabilization, primarily requires properly folded zinc fingers (8,41,42). In the case of HIV-1 NC, this activity depends on the preferential binding of the zinc fingers to unpaired NA bases (43,44). The duplex destabilization activity differs significantly between different retroviral NC proteins, as well as related retrotransposons (6,45–47).

Although many aspects of NC’s chaperone activity have been extensively investigated, the detailed relationship between HIV-1 NC’s structure and its ability to aggregate and destabilize NAs is still incompletely understood. For example, although the effective cationic charge of HIV-1 NC, defined as a negative slope of the log-log dependence of the *K_d_* versus salt concentration, is ∼+3.5 (48–52), the total number of positively charged residues on this protein is 15, with only four negatively charged residues. Thus, it is unclear how and to what extent specific HIV-1 NC charged residues participate in nonspecific versus specific NA binding. The role of specific basic residues in other chaperone activities, such as NA aggregation, is also unknown.

To clarify the interplay between specific basic residues and HIV-1 NC’s NA chaperone function, we use several complementary *in vitro* approaches. Ensemble assays are used to quantify NA binding, aggregation and annealing activities of wild-type (WT) and mutant HIV-1 NC proteins (6,8). These results are compared with the results obtained from single-molecule DNA stretching experiments. We find that cationic HIV-1 NC variants are defective in their overall NA binding affinity, aggregation and strand-annealing activities, but retain significant NA stacking capability at sufficiently high concentrations. These results are in contrast to previous work showing that aromatic residue variants are completely defective in stacking with NA bases, and have greatly reduced single-stranded DNA (ssDNA) binding affinity (8). Cell-based assays showed that all of the cationic residue mutations investigated lead to reduced virus infectivity, which correlated strongly with measurements of trans-activation response (TAR) RNA and DNA annealing as well as the capability to intercalate DNA at high force in single-molecule stretching experiments.

## MATERIALS AND METHODS

### Plasmids, mutagenesis and recombinant protein production

**Figure 1.**
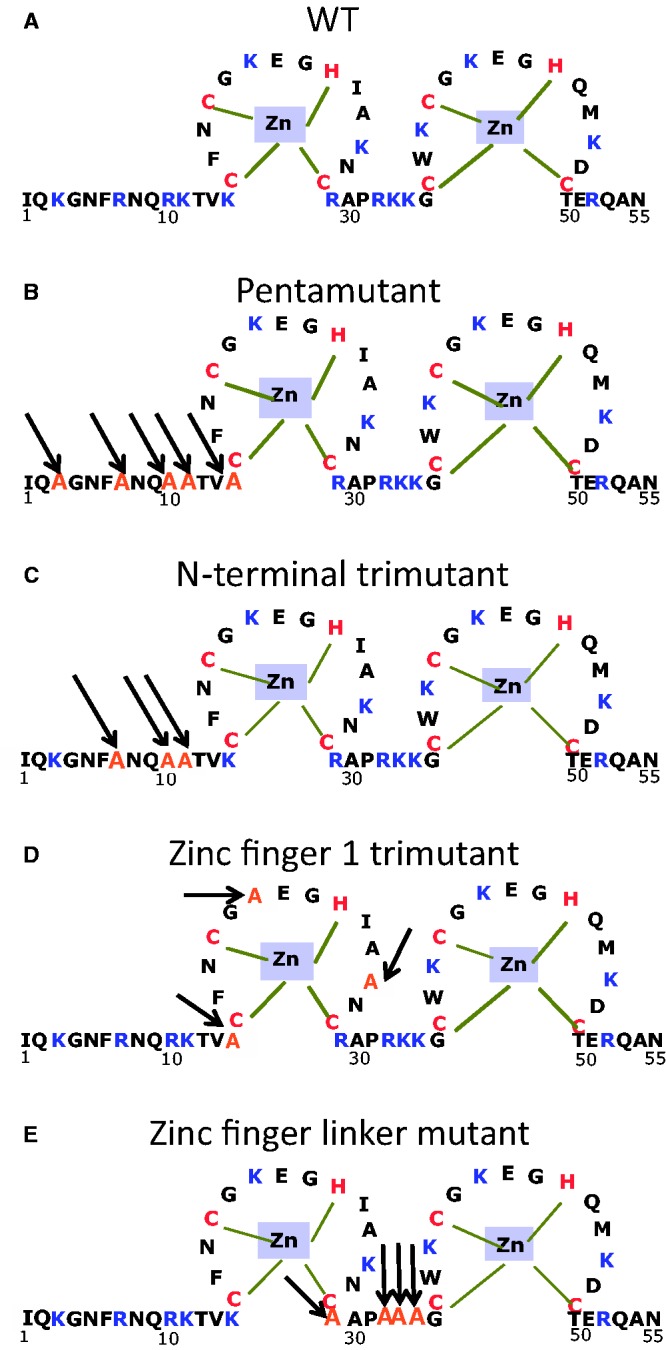
Sequence of WT HIV-1 NC (NL4-3 isolate) and variants investigated in this work. (**A**) WT; (**B**) K3A/R7A/R10A/K11A/K14A (pentamutant); (**C**) R7A/R10A/K11A (N-terminal trimutant); (**D**) K14A/K20A/K26A (Zinc finger 1 trimutant); (**E**) R29A/R32A/K33A/K34A (Zinc finger linker mutant).

Site-directed mutagenesis was performed using the Agilent QuickChange kit, with verification by NA sequence analysis, for the generation of full-length proviral pNL4-3 plasmids [pNL4-3, GenBank accession no. AF324493 was obtained through the AIDS Research and Reference Reagent Program, Division of AIDS, NIAID, NIH from Dr Malcolm Martin (53)] or recombinant expression plasmids essentially as described (8). Mutations are as follows with numbering of nucleotide positions based on the pNL4-3 clone of HIV-1 (GenBank accession no. AF324493): K3A changes nucleotide 1927 to 1928 from aa to gc, R7A changes nucleotide 1939 to 1940 from ag to gc, R10A changes nucleotide 1948 to 1949 from ag to gc, K11A changes nucleotide 1951 to 1952 from aa to gc, K14A changes nucleotide 1960 to 1961 from aa to gc, K20A changes nucleotide 1978 to 1980 from aaa to gcc, K26A changes nucleotide 1996 to 1997 from aa to gc, R29A changes nucleotide 2005 to 2007 from agg to gca, R32A changes nucleotide 2014 to 2016 from agg to gcc, K33A changes nucleotide 2017 to 2019 from aaa to gcc, K34A changes nucleotide 2020 to 2022 from aag to gcc. All mutations were verified by NA sequence analysis. Recombinant NC proteins, 55 amino acids in length, were expressed and purified as described (33,45,54). The mutant NC amino acid sequences examined in either viruses or as purified NCp7 are presented in Figure 1.

### Single-molecule DNA stretching studies

In the single-molecule stretching experiments performed essentially as described (55), two laser beams are overlapped to trap one streptavidin-coated bead. A second streptavidin-coated bead is attached to a glass micropipette. Bacteriophage λ DNA, which was labeled with biotin on both 5′ ends as described (56), is caught between the beads due to strong noncovalent bonds between biotin and streptavidin. All stretching experiments were performed at a constant pulling rate of ∼100 nm/s in 10 mM HEPES, 50 mM Na^+^, pH 7.5 buffer. After attachment of one DNA molecule, buffer was used to rinse out the other DNA molecules, and solutions containing specific protein concentrations were flowed around the DNA to investigate protein effects on DNA stretching curves.

### Ensemble studies

#### Fluorescence anisotropy binding studies

Twenty nanomolar 5′ fluorescein-labeled 18-mer microTAR RNA (8) (Dharmacon, Inc.) or 10 nM 5′-carboxyfluorescein (FAM)-labeled-(TG)_4_ DNA (8) (Integrated DNA Technologies, Coralville, IA) was incubated in the absence or presence of varying concentrations of WT or mutant NC proteins at 20°C in 20 mM HEPES, pH 7.5, 50 mM NaCl, 10 µM tris (2-carboxyethyl)phosphine, 5 mM β-mercaptoethanol and 1 µM zinc acetate for 30 min. Fluorescence anisotropy (FA) measurements were carried out in duplicate for each protein concentration using 20 µl of solution per measurement. Assays were performed in Corning® 384-well low volume polystyrene NBS™ microplates (Corning, NY) using a SpectraMax® M5 multimode microplate reader (Molecular Devices, Sunnyvale, CA). Excitation and emission wavelengths were set at 485 nm (9 nm bandwidth) and 525 nm (15 nm bandwidth), respectively. Anisotropy (A) was calculated using SoftMax® Pro (Molecular Devices) software. The resulting plot of A versus NC concentration (N) was fit using Kaleidagraph (Synergy Software, Reading, PA) according to the following one-site binding model (56):
(1)
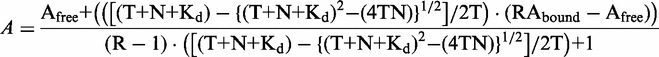

where T, A_bound_, A_free_, K_d_ and R represent the concentration of oligonucleotide, anisotropy of completely bound oligonucleotide, anisotropy of unbound oligonucleotide, equilibrium dissociation constant and the ratio of fluorescence intensity of completely bound oligonucleotide relative to unbound nucleotide, respectively. The anisotropy of completely bound oligonucleotide was determined as a fitting parameter representing saturated binding for each measurement.

#### TAR RNA/DNA annealing assays

Annealing assays were carried out with 59-nt TAR RNA and 59-nt complementary TAR (cTAR) DNA as described previously (8). Immediately prior to each experiment, ^32^P-labeled TAR RNA (1.5 µM) and cTAR DNA (6 µM) solutions were refolded in 25 mM HEPES, pH 7.5, and 20 mM NaCl by heating at 80°C for 2 min, then cooling to 60°C for 2 min, followed by addition of MgCl_2_ (to 10 mM final) and placement on ice. For annealing assays, ^32^P-labeled TAR RNA (15 nM) and cTAR DNA (45 nM) were combined in a solution containing 20 mM HEPES, pH 7.5, 20 mM NaCl, 0.2 mM MgCl_2_ and 5 mM dithiothreitol (DTT). The solution was incubated at 37°C for 5 min before addition of NC to a final concentration of 0.88 µM, corresponding to a 4:1 nt:NC ratio, unless otherwise indicated. At desired time points, an aliquot of the reaction mixture was quenched by addition of 1% sodium dodecyl sulphate, followed by incubation at room temperature for 5 min and placement on ice. The aliquots were phenol-chloroform extracted twice (to remove any NC bound to the NA) followed by addition of glycerol (5%) and separation on 12% polyacrylamide gel electrophoresis (19:1 acrylamide:bisacrylamide). The gels were visualized using a Bio-Rad (Hercules, CA) Molecular Imager FX and quantified with Bio-Rad Quantity One Software.

#### Aggregation assays

Sedimentation assays to monitor NC-induced NA aggregation activity were performed as previously described (36). Briefly, TAR RNA (unlabeled) was combined with ^32^P-labeled TAR RNA to a final concentration of 1.5 µM. Both ^32^P-labeled TAR RNA and cTAR DNA (6 µM) were folded as described for annealing assays (see below) and diluted to a final concentration of 15 and 45 nM, respectively, in a buffer containing 20 mM HEPES, pH 7.5, 20 mM NaCl, 0.2 mM MgCl_2_, and 5 mM DTT in the absence or presence of 0.88 µM NC, unless otherwise indicated. The samples (30 µl) were incubated at 37°C for 30 min and then spun in a Micromax RF (Thermo IEC) centrifuge rotor at a speed of 12 000 rpm for 10 min at 4°C. An aliquot (4 µl) of supernatant was taken and the amount of radioactivity measured using scintillation counting.

#### Cell culture-based studies

Full-length mutant or WT proviral plasmids were transfected into 293T cells and viruses were harvested as described previously (57). Viral genome packaging was assessed by measuring gRNA by quantitative reverse transcriptase-polymerase chain reaction and normalizing to exogenous-template reverse trancriptase (RT) activity as described (57). Single-round infectivity assays using HCLZ or TZM-bl cells as well as multiple-round replication assays using H9 cells were performed as described (57,58).

#### Correlation measurements

Correlation coefficients were calculated according to the following equation:
(2)
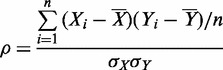

where *ρ* is the correlation coefficient, 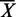 and 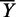 are the average values of 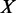 and 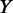, 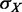 and 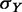 are the standard deviations of 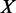 and 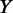 (59).

## RESULTS

### DNA stretching in the presence of WT HIV-1 NC

**Figure 2.**
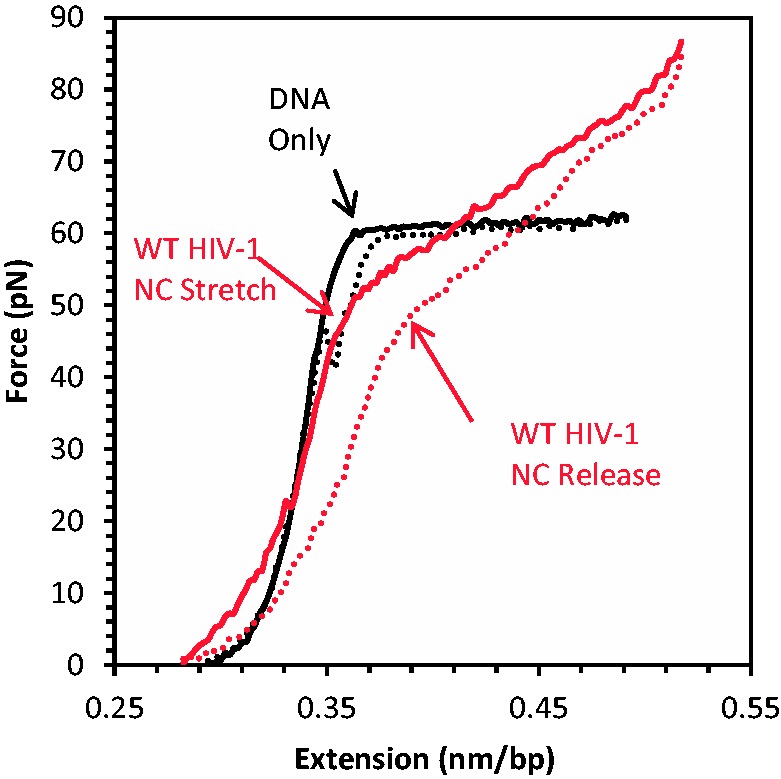
Force-extension (solid) and release (dashed) curves of DNA alone (black) and in the presence of 30 nM HIV-1 NC (red). Adapted from reference (8).

Figure 1 shows the sequences of WT HIV-1 NC and the basic residue variants studied in this work. Single-molecule λ-DNA stretching studies were carried out as previously described (8). As shown in Figure 2, in the absence of protein, little force is required to stretch the DNA to its B-form contour length, which is ∼0.34 nm/bp. As the contour length is approached, the force increases dramatically, reflecting the elasticity of the DNA helix. As the DNA is further stretched, it undergoes a force-induced overstretching transition from double-stranded DNA (dsDNA) to ssDNA. During this process, the DNA extension increases from 0.34 nm/bp to 0.6 nm/bp at an approximately constant force of 60 pN. The overstretched state of DNA can be either completely strand-separated with one or both strands under tension, or stretched to an unwound but still double-stranded structure, depending on solution conditions, DNA sequence and pulling rate (60–64). Under conditions of relatively low solution ionic strength as used here, or in the presence of ssDNA binding proteins, the DNA becomes single-stranded during the transition (62,63). In contrast, in the presence of proteins or ligands that preferentially bind to both DNA strands, the two strands may never separate. In the absence of protein, as the DNA is released back to lower extension, the force-extension curve is almost completely reversible, showing little hysteresis.

In the presence of WT HIV-1 NC, the overstretching transition becomes dramatically sloped, as the DNA starts to elongate beyond its B-form contour length from 20 to 30 pN (16,56) (Figure 2). The transition in this case is poorly defined, and extends over a broad range of forces between 20 and 90 pN, leading to an apparent force-extension slope up to 350 pN/nm/bp (Figure 2). A similar effect on DNA stretching was previously observed in the presence of intercalators such as ethidium (65), several ruthenium complexes (66,67) and threading- and bis-intercalators (68). In contrast to NC, these intercalators bind preferentially to dsDNA and increase the overall stability of duplex DNA, and this is usually reflected in an overall increase in the DNA stretching force. An example of a destabilizing intercalator is the anticancer drug Actinomycin D (Act D), which was recently characterized by DNA stretching (69). The stretching curves in the presence of Act D strongly resemble those observed in the presence of NC. This observation, when combined with our recent observation that the two DNA strands do not separate on overstretching in the presence of NC (8), suggests that NC acts as a weak intercalator, which on binding holds the two DNA strands together while simultaneously destabilizing the duplex form. Thus, the significant DNA elongation observed in the presence of NC at forces >20 pN results from the stacking of some NC residues between dsDNA bases, resulting in an intercalation process, similar to that observed for Act D (69), although it occurs at a much faster rate. In the case of HIV-1 NC, this duplex elongation likely occurs on intercalation of the aromatic residues (Phe16 and Trp37) (70,71), similar to the stacking with bases that was observed in the nuclear magnetic resonance (NMR) structures of HIV-1 NC bound to HIV-1 stem loops SL2 and SL3 (43,44,51,72–79). Here, we will use the value of the slope as a primary quantitative characteristic of NC-DNA binding, which reflects the ability of these aromatic residues to optimally stack with the DNA bases.

### DNA stretching with HIV-1 NC basic residue mutants

**Figure 3.**
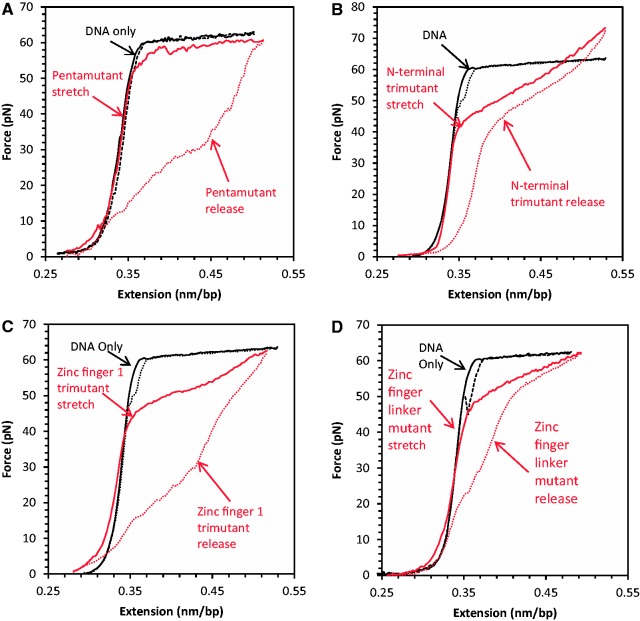
DNA stretching (solid line) and release (dotted line) in the presence (red) and absence (black) of HIV-1 NC cationic mutants: (**A**) 200 nM N-terminal pentamutant (K3A/R7A/R10A/K11A/K14A); (**B**) 200 nM N-terminal trimutant (R7A/R10A/K11A); (**C**) ZF-1 cationic mutant (K14A/K20A/K26A); (**D**) Zinc finger linker mutant (R29A/R32A/K33A/K34A).

Presented in Figure 3 are the stretch and release curves obtained in the presence of HIV-1 NC basic residue mutants. All of these curves differ significantly, showing high sensitivity to even a few amino acid substitutions. To summarize the protein–DNA interaction information contained in these data, we use two quantitative parameters describing the stretch–release cycle for the protein–DNA complex: the slope of the overstretching transition, *S*, and the scaled hysteresis in the stretch–release cycle, *H*, which is derived from the area between the stretch and release curves (8). *S* is measured as the slope of the tangent line to the force-extension curve at the transition midpoint of the extension (∼0.48 nm/bp). The protein-free slope, *S_0_*, is ∼20 pN/nm/bp, and the protein-saturated maximum slope, *S_max_*, reflects the protein’s ability to intercalate into dsDNA on saturated binding at high forces. A small transition slope close to *S_0_* indicates a force-induced DNA strand separation. In contrast, *S_max_>>S_0_* reflects strong intercalation by the protein at high force. The theoretical maximum amount of hysteresis would be achieved if DNA could be stretched as dsDNA and released as ssDNA. We define the scaled hysteresis, *H*, as the actual area of the protein–DNA stretch–release cycle divided by this maximum possible value. If the protein intercalates DNA, this may result in small *H* because it prevents the two strands from separating when being stretched. If the protein does not intercalate DNA and the strands are separated by force during overstretching, then small *H* indicates that the strands rapidly anneal on the time scale of stretching. Larger values of H may occur if the DNA intercalates but the intercalation is slow, as is the case for Act D. If there is no intercalation and the strands separate during stretching, large hysteresis may result from protein binding to ssDNA and subsequent slow dissociation. We will use these observed features of DNA stretching curves below to probe the effects of cationic mutations on NC–DNA interactions.

#### Effect of HIV-1 NC basic residue mutations

Presented in Figure 3A is a typical DNA stretch–release cycle in the presence of an HIV-1 NC variant with 5 N-terminal basic residues changed to alanine (K3A/R7A/R10/K11A/K14; pentamutant) (see Figure1B). Surprisingly, the dsDNA stretching curve in the presence of 200 nM pentamutant resembles the curve in the absence of protein. The fact that the transition slope remains close to the protein-free value (Figure 3A) suggests that the protein does not intercalate DNA and the DNA is melted by force in the presence of the pentamutant NC, consistent with the large hysteresis observed on DNA release. The large hysteresis also suggests strong and relatively slow binding of the pentamutant to ssDNA. It is initially surprising that the pentamutant NC is unable to intercalate DNA, as there is still an aromatic residue on each of the two zinc fingers. However, it has been shown that the strength of DNA intercalation by proteins depends strongly on interactions with amino acid side chains that are outside the intercalative wedge of the protein, and this must be the case for NC as well (80). In addition, it is clear that the intercalative ability of the pentamutant is weakened but not eliminated, as the DNA stretching slope increases with concentration.

**Figure 4.**
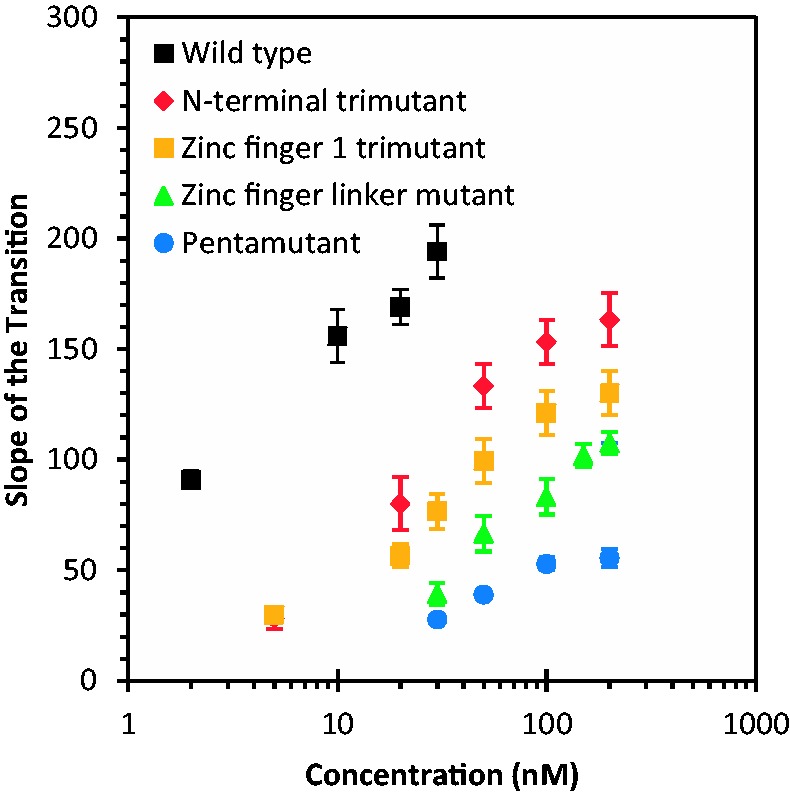
Slope of the transition versus concentration for WT HIV-1 NC and cationic mutants. Note that the 5 nM data points for the zinc finger 1 linker mutant and the N-terminal tri-mutant are coincident.

**Figure 5.**
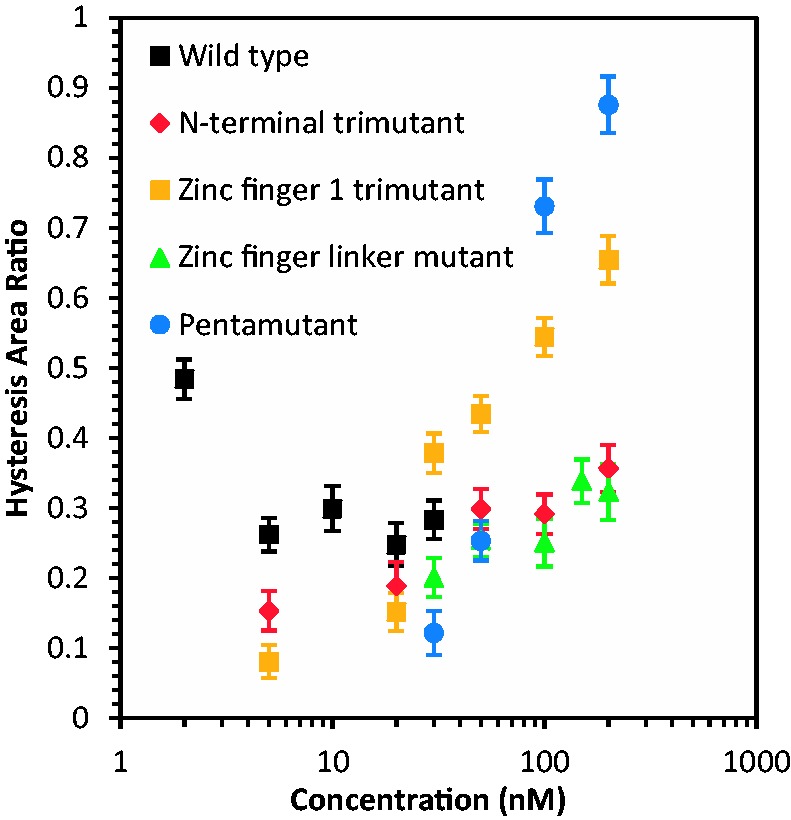
Hysteresis ratio versus concentration curves for WT HIV-1 NC and cationic mutants.

Figure 3B shows the result of the DNA stretch–release curves in the presence of an N-terminal NC variant with only three of the basic residues mutated to alanine (R7A/R10A/K11A; N-terminal trimutant). This variant displayed a greatly increased *S* relative to the pentamutant, but a reduced *H*, suggesting efficient DNA intercalation by the N-terminal trimutant, similar to that observed for WT NC. Two additional basic residue mutants containing three (K14A/K20A/K26A; zinc finger 1 trimutant) or four (R29A/R32A/K33A/K34A; zinc finger linker mutant) changes displayed features suggesting weakened intercalative binding by these proteins as well as partial DNA melting by force, as determined by the smaller slopes and larger hysteresis observed compared with WT or N-terminal trimutant NC (Figure 3C and D). A summary of the *S* and *H* parameters calculated for all the cationic NC mutants investigated as a function of protein concentration is presented in Figures 4 and 5, respectively. For all mutants, the *S* parameter increases with concentration, suggesting that the ability to intercalate is strongly enhanced by binding that is facilitated by basic residues, and this enhancement is different depending on the location and number of the residues. The *H* parameter also increases with concentration for all basic residue mutants studied, in contrast to WT NC (Figure 5). This result reflects the loss of the ability of all of these mutants to intercalate DNA, along with defects in their abilities to aggregate ssDNA and promote strand annealing after separation by force. Based on these results, we can conclude that changes to any cluster of basic residues in the N-terminus, N-terminal zinc finger or linker region of HIV-1 NC significantly impacts at least one of these characteristics, which are associated with NA chaperone activity.

The fact that all of these mutations alter DNA interactions is notable, as the net effective charge of WT HIV-1 NC binding to NA is only ∼+3.5, which is much smaller than the total number of cationic residues (+15) (48–52) distributed throughout the protein. The effective charge implies that ∼3.5 Na^+^ ions are released from the NA on HIV-1 NC binding. The fact that mutation of several different subsets of cationic residues along the protein sequence affects the binding implies that a majority of the cationic residues participate in direct interactions with the NA phosphates, but to different extents. Thus, the extent to which mutations contribute to HIV-1 NC-DNA interaction defects does not completely correlate with the total number of altered basic residues (3, 4 or 5). The quantity most reflective of the ability of NC to intercalate DNA, *S*, is greatest for the least number of residues changed (N-terminal trimutant) and lowest for the greatest number of residues changed (pentamutant). The N-terminal trimutant and the zinc finger 1 trimutant exhibit different slopes within uncertainty, despite having the same number of basic residues mutated. These observations illustrate the differential contributions of the cationic mutations to the ability of NC to intercalate. These results are consistent with the effects observed for cationic mutations on high mobility group proteins and other intercalating proteins (80).

### Ensemble studies of HIV-1 NC basic residue variants

#### FA binding studies

We next examined the effect of basic residue mutations on binding to two oligonucleotides using FA. We chose an 18-nt RNA derived from TAR (micro-TAR RNA) and a ssDNA sequence known to be a preferred NC-binding substrate, the 8-nt (TG)_4_ oligomer (82). Relative to WT HIV-1 NC, which bound to micro-TAR and (TG)_4_ with relatively high affinity (315 and 5 nM K_d_, respectively), the pentamutant bound with 8- and 12-fold reduced affinity to these oligonucleotides, respectively (Table 1). The N-terminal trimutant was less defective in NA binding than the pentamutant, displaying 5-fold lower affinity binding to both oligonucleotides (Table 1). The zinc finger 1 mutant only displayed 2- to 4-fold lower affinity binding to micro-TAR RNA and (TG)_4_, respectively. The linker mutant resulted in the largest binding defect to (TG)_4_ (16-fold) but only a <2-fold decreased affinity to micro-TAR. Overall, the cationic mutations in NC significantly alter binding to both nucleotide sequences. Interestingly, mutations of NC’s aromatic residues resulted in poor binding to the ssDNA sequence (TG)_4_, while the binding of aromatic residue mutants to micro-TAR RNA was only marginally affected (8). These results are consistent with the hypothesis that the cationic residues of NC are responsible primarily for the nonspecific electrostatic binding to NAs. This nonspecific binding is strongest for double-stranded NA molecules, while the aromatic residues in the zinc fingers of NC are responsible for the specific stacking interactions involved in binding to TG-rich single-stranded sequences (51,82,83).

**Table 1.** Binding, aggregation and annealing parameters measured for WT HIV-1 NC and basic residue mutants

HIV-1 NC variant	Micro-TAR RNA^a^ *K_d_* (nM)	(TG)_4_ DNA^a^ *K_d_* (nM)	%TAR RNA aggregated^b^	TAR RNA/DNA annealing rate^c^ min^−1^
WT	315 ± 48	5 ± 1	94.2 ± 1.2	>6
N-terminal trimutant	1720 ± 129	27 ± 2	84.7 ± 3.1	1.19 ± 0.56
ZF1 trimutant	591 ± 138	19 ± 10	93.8 ± 1.5	0.51 ± 0.38
ZF linker mutant	552 ± 20	82 ± 54	79.3 ± 9.5	0.60 ± 0.41
Pentamutant	2440 ± 370	62 ± 20	85.4 ± 2.5	0.09 ± 0.07

^a^Apparent equilibrium dissociation constants, *K_d_*, for binding to NA oligomers measured at room temperature in 50 mM NaCl by FA as described in ‘Materials and Methods’ section.

^b^Percent TAR RNA aggregated by 10 µM protein in a solution containing 15 nM radiolabeled TAR RNA and 45 nM TAR DNA at 37°C in 50 mM NaCl.

^c^TAR RNA-DNA annealing rate in the presence of 10 µM protein under the same solution conditions used in the aggregation assay.

#### NA annealing

**Figure 6.**
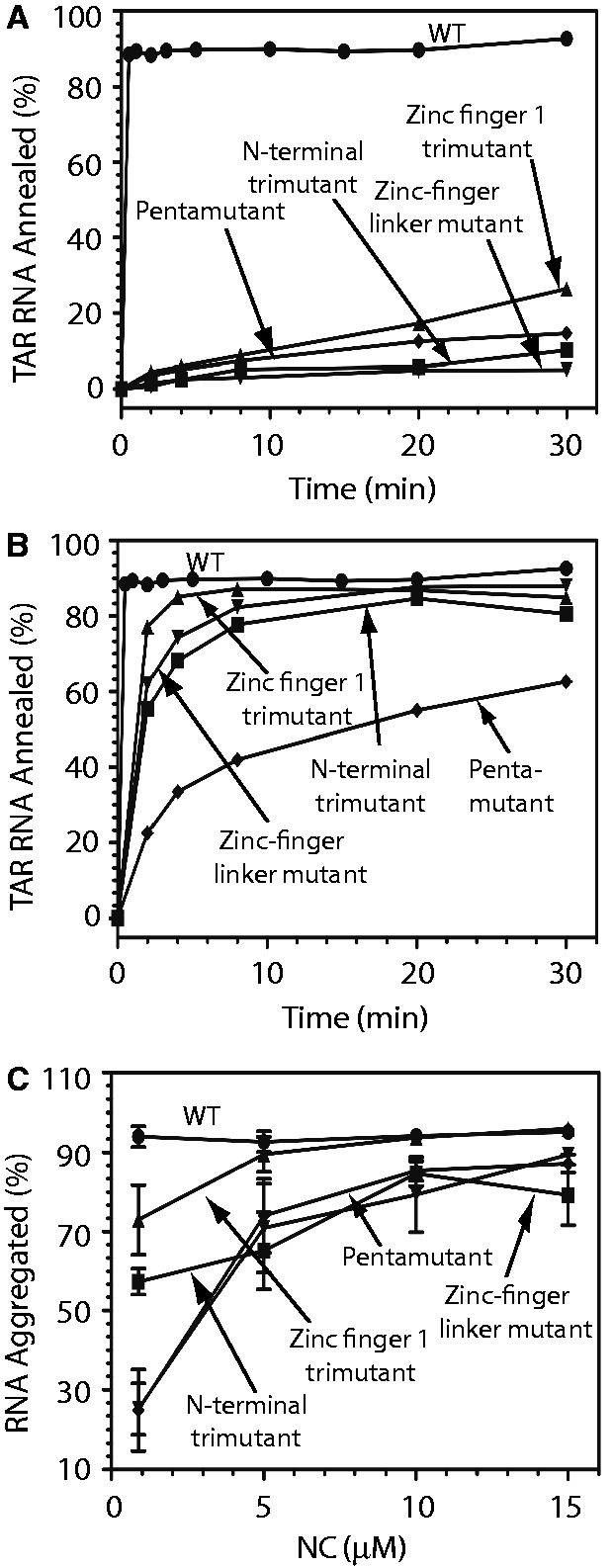
Percent TAR RNA annealed to TAR DNA (**A**, **B**) or aggregated (**C**) by WT or mutant HIV-1 NC. Annealing assays were conducted using 0.88 µM WT or mutant NC (panel A) or 0.88 µM WT and 10 µM mutant NC (panel B). Aggregation assays were performed as a function of protein concentration as indicated.

To assess the cationic HIV-1 NC mutants in chaperone function, we performed gel-shift TAR RNA/DNA annealing assays as previously described (8). This assay requires both aggregation and destabilization activity and is therefore a good indicator of overall chaperone function. When low amounts of NC were used [0.88 µM or 1 NC per 4 nt (36)], the WT annealing reaction proceeds with a rate of >∼6 s^−^^1^, showing >10^4^-fold annealing rate enhancement over the background rate of annealing in the absence of protein. None of the four cationic HIV-1 NC mutants studied in this work were able to facilitate the annealing kinetics to any measurable degree when added at this concentration (Figure 6A). However, at high concentrations (10 µM), all of the NC basic residue variants facilitated annealing, but to varying extents (Figure 6B). The fitted values of the TAR RNA/DNA annealing rates under these conditions are summarized in Table 1. As 10 µM significantly exceeds the measured K_d_ values for any of these cationic HIV-1 NC mutants binding to either double-stranded or single-stranded NA, the differences in their ability to facilitate TAR annealing reflects differences in chaperone function.

#### NA aggregation

The effect of basic residue mutations on NA aggregation is summarized in Figure 6C. All cationic HIV-1 NC mutants displayed reduced NA aggregation activity relative to WT HIV-1 NC, as expected from their reduced NA binding affinity. The effect generally increased with increasing numbers of basic residues mutated. Substitution of the three basic residues in the N-terminus (trimutant) had a greater effect on RNA aggregation than neutralizing the three basic residues in zinc finger 1. The linker mutant and pentamutant were the most defective at low NC concentrations but were almost as effective at NA aggregation as WT NC at 10 µM concentration (Table 1). NC aggregates all NA nonspecifically through an electrostatic mechanism that is due to its overall high charge density (6,12,13,19,45). This electrostatic attraction mechanism is similar to that observed for multivalent cations of charge ≥+3 (84–87). The fact that NC mutants with 3 to 5 positive charges neutralized still retain the ability to aggregate NA at saturated binding illustrates the adjustable nature of NC–NA interactions. Here it appears that the role of the neutralized residues can be replaced by the remaining positive charges distributed through the rest of the molecule.

### Cell culture-based studies

Table 2 summarizes the effects of the NC basic residue mutations expressed in HIV-1 virions on gRNA packaging, and single- and multiple-round infectivity. Single-round infectivity levels reflect the combined effect of the mutations on the ability of virions to ultimately produce the Tat protein, resulting in positive events in the HCLZ or TZM-bl cells used (58). The fractional gRNA packaging levels reported in the second column of Table 2 show relatively minor defects for all but the pentamutant NC, which only packages ∼3% WT-levels of gRNA. Packaging levels for the zinc finger 1 trimutant are in agreement with the previous study by Poon and Aldovini (88). However, in all cases, single-round infectivities (column 3) are more defective than genome packaging, with the pentamutant being considerably more defective in single-round replication. These defects are compounded in subsequent replication cycles, leading to exponential defectiveness reflected in the multiple-round infectivity assay data in the far right column of Table 2. Although the uncertainties for multiple-round infectivity are close to the mean, the overall measurements vary by orders of magnitude for different mutants. Thus, reduction of the basic character of NC affects genome packaging but more significantly disrupts reverse transcription processes (discussed below) as observed by (i) a reduction in Tat-mediated focus forming activity in single-round assays and (ii) severe reductions in multiple-round replication.

**Table 2.** Properties of WT HIV-1 NC and basic residue mutants measured in cells

NC protein	RNA packaging^a^	Single-round infectivity^b^	Relative multiple-round H9 infectivity^c^
WT	100	100	1.0
N-terminal trimutant	51 ± 19	7.4 ± 1.6	(3.8 ± 3.3)×10^−3^
Zinc finger 1 trimutant	31 ± 14	8.8 ± 2.4	(5.0 ± 4.4)×10^−5^
Zinc finger linker mutant	61 ± 11	3.2 ± 0.7	(7.2 ± 4.5)×10^−5^
Pentamutant	3.0 ± 0.3	0.016 ± 0.007	≤(7.1)×10^−6^^d^

^a^Determined by normalizing genome quantities to equivalent RT activities and reported as % of WT. Results from at least two separate experiments with standard deviations reported.

^b^Either HCLZ or TZM-bl cells were used for these analyses. Titers (reported as % of WT) were determined by taking the BCFU/ml of the mutant and dividing by WT titer, corrected for input virus (based on exogenous template RT activity). Results are from at least four separate experiments. Errors are standard deviations.

^c^Determined by taking the minimum dilution that gives rise to a spreading infection over 8 weeks (average of at least three infection experiments), normalized for equivalent exogenous template RT activities. Titers of mutants reported relative to a WT infection. Errors represent the standard error of the mean. The uncertainties are close to the mean due to the choice of dilutions used.

^d^Three independent analyses were performed with the undiluted sample of the pentamutant being negative in each assay.

To understand how the observed biophysical effects contribute to viral infectivity and RNA packaging, we calculated the correlation coefficient between several of the *in vitro* measurements and measurements in cells. The results can be found in Table 3. While all of the *in vitro* measurements show some correlation with RNA packaging and replication in cells, only the TAR RNA/DNA annealing and single-molecule measurements of the DNA overstretching slope show overall strong correlations.

**Table 3.** Correlation of *in vitro* WT and mutant HIV-1 NC measurements with cellular replication and packaging measurements for the same mutants

Correlation	RNA packaging	Single-round infectivity	Log (multiple-round infectivity)
*K_d_*_,_ microTAR RNA	−0.74	−0.53	−0.48
*K_d_*_,_(TG)_4_ DNA	−0.42	−0.65	−0.69
RNA/DNA annealing rate	0.85	0.99	0.94
DNA stretching hysteresis	−0.91	−0.52	−0.76
Transition slope	0.89	0.81	0.96

Correlation coefficients are determined from Equation (2), using values from Table 1 for *K_d_* or annealing rate and Table 2 for measurements in cells. Single molecule transition slope and hysteresis were evaluated at 200 nM NC for the mutants and 20 nM for WT NC. To take into account the exponential effect of multiple rounds of replication, we used the logarithm of the infectivity for correlation calculations. Positive results mean that the two quantities are correlated and negative results mean inverse correlation. Assuming a one-tailed distribution, for five measurements the correlation must be greater than 0.805 for 95% confidence in the correlation, and 0.687 for 90% confidence (59). Therefore, correlations >0.69 should be considered strong for this number of measurements.

## DISCUSSION

In this work, we study the effect of HIV-1 NC cationic mutants on protein–NA interactions and retroviral replication. All of the basic residue mutations that were tested reduced micro-TAR RNA binding affinity, with the pentamutant showing the greatest reduction in affinity, as expected. However, the N-terminal trimutant exhibits the strongest reduction in binding relative to the number of residues changed. In contrast, the strongest reduction in binding affinity to (TG)_4_ DNA comes from the zinc finger linker mutant and the pentamutant, with the linker mutant having the strongest effect per residue mutated. Therefore, the ability of NC to bind short NA sequences decreases with the number of neutralized basic residues, but the positions of these residues are also important. These results suggest that the cationic residue mutations on the N-terminal domain most strongly alter the binding affinity to nonspecific NA sequences, represented by the micro-TAR RNA, while mutations on the zinc finger linker have a greater effect on the protein’s ability to stack with NAs, as required for optimal binding to (TG)_4_. Although all of the mutants aggregated NA at high concentration, they were less effective aggregating agents at the lowest concentration tested (0.88 µM) and the percent aggregated even at 10 µM was somewhat less for all of the mutants except the zinc finger 1 mutant.

The single-molecule studies also revealed defects in DNA interactions due to basic residue mutations. For all of the mutants, the ability of NC to alter the slope of the DNA stretching curve was significantly compromised, but the slope change was recovered to some extent by increasing the concentration of protein used. The ability to alter the stretching slope was weakest for the pentamutant, followed by the zinc finger linker mutant, the zinc finger 1 trimutant and finally the N-terminal trimutant, which was closest to WT NC activity. However, while these effects increase with the number of residues mutated, the zinc finger 1 and N-terminal trimutants exhibit significantly different effects, supporting the conclusion that the positions of the residues that are mutated are critical. Similarly, the amount of hysteresis measured for the mutants also increases with the number of residues mutated, with the hysteresis area ratio at 200 nM being greatest for the pentamutant. However, in this case the zinc finger 1 trimutant is almost as defective as the pentamutant, and the other mutants are closer to WT NC. Overall, the single molecule studies show that each set of mutated residues decreases NC’s ability to optimally interact with DNA to varying extents, depending on the location of the mutated residues.

To determine the extent to which the results of these *in vitro* measurements reflect the ability of NC to facilitate viral replication in cells, we calculated the correlation coefficient between the *in vitro* single molecule, binding and annealing measurements and measurements of RNA packaging, single-round infectivity and multiple-round infectivity in cells (Table 3). We have also plotted the measurements in cells as a function of the *in vitro* measurements, along with their linear fits, and these results are shown in Supplementary Figures S1 and S2. Because cationic residue mutations are expected to primarily reduce electrostatic binding interactions, it would be reasonable to expect the cell-based measurements to result in a negative correlation coefficient with *K_d_*. Surprisingly, however, the measurements in cells correlate only moderately with binding affinity to microTAR or (TG)_4_ DNA, with the absolute value of these correlation coefficients less than the 90% confidence interval for two of the three measurements, and less than 95% confidence for correlation with all three. Visual examination of the plots in Supplementary Figure S1 (a-f) confirms that there is not a strong correlation between *in vitro* binding affinity and measurements in cells, although removal of the WT data point from Supplementary Figure S1 (e) results in a strong negative correlation between the binding affinity of mutant NC to (TG)_4_ DNA and single-round infectivity. The lack of strong correlation between the binding of the cationic NC mutants and the extent of RNA packaging and replication defects observed in cells is consistent with the hypothesis that the aromatic zinc finger residues play the primary role in gRNA selection and chaperone function, while the cationic residues play only a secondary role in these functions. This result is also consistent with the hypothesis that the chaperone activity of NC occurs *in vivo* when NC concentrations are in excess of the *K_d_* values observed here even for the most binding-defective cationic NC mutants (Table 1). For example, cumulative evidence suggests that HIV-1 reverse transcription is mechanistically linked to CA uncoating (89–98) and the early steps of reverse transcription are likely to occur within an intact CA core.

The ability of NC to facilitate TAR RNA/DNA annealing appears to correlate strongly with RNA packaging and replication measurements based on Table 3, consistent with the importance of overall NA chaperone activity for viral replication (8). However, because the results of the TAR RNA/DNA annealing measurements for WT protein are significantly different than those of the mutants, this leads to an anomalously high correlation between annealing and measurements in cells, as shown in Supplementary Figure S1 (g–i). When the WT NC data point is removed from these graphs, the correlation coefficients become 0.75, 0.66 and 0.99 for correlation of annealing rate with RNA packaging, single-round infectivity and the logarithm of multiple-round infectivity, respectively. This suggests a moderate to strong correlation between TAR RNA/DNA annealing and replication measurements in cells. Similarly, the ability of NC to prevent DNA strand separation by force, as reflected in small hysteresis, also correlates strongly with RNA packaging, and moderately with multiple-round infectivity (Table 3, DNA stretching hysteresis). The hysteresis measurements do not change significantly when influential data points are removed. In addition to RNA/DNA annealing, the other strong correlation between *in vitro* measurements and measurements in cells is in the slope of the single-molecule DNA stretching curve. All three measurements in cells correlate strongly with the slope *S*, even when influential data points are removed. As discussed above, this change in slope reflects the ability of the protein to elongate dsDNA without allowing the strands to separate. This elongation at high forces represents a measurement of DNA intercalation by NC, which only occurs at high forces. However, the correlation with replication measurements suggests that this optimal ability to intercalate is a critical component of NC’s NA chaperone activity. Similar stacking interactions between the F16 and W37 residues of NC and single-stranded bases were recently characterized by NMR (79). These interactions resemble the hemi-intercalation observed between the aromatic rings of the dsDNA intercalator Act D and ssDNA bases (99). While the observed intercalation is too weak to occur frequently at zero force when NAs are fully double-stranded, the intercalation measured in these single-molecule experiments likely becomes important in cases of locally unstable elements of NA secondary structure, such as duplexes containing mismatches, loops or bulges. Thus, these combined experiments demonstrate a strong correlation between *in vitro* NA chaperone activity and cellular replication measurements, illustrating the importance of specific HIV-1 NC basic residues for these processes.

## SUPPLEMENTARY DATA

Supplementary Data are available at NAR Online.

## FUNDING

Federal funds from the National Cancer Institute, National Institutes of Health, under contract HHSN261200800001E with Leidos Biomedical Research, Inc. (RJG); National Institutes of Health [GM065056 to K.M.-F. and GM072462 to M.C.W.]; National Science Foundation [MCB-1243883 to M.C.W]. Funding for open access charge: National Institutes of Health.

*Conflict of interest statement*. None declared.

## Supplementary Material

Supplementary Data
