# Supplementary material for: Differential contribution of basic residues to HIV-1 nucleocapsid protein’s nucleic acid chaperone function and retroviral replication
Source: Nucleic Acids Res. 2013 Nov 28;42(4):2525–37. doi: 10.1093/nar/gkt1227 (PMC3936775; doi:10.1093/nar/gkt1227)
Supplement: Supplementary Data [file supp_42_4_2525__index.html]

Differential contribution of basic residues to HIV-1 nucleocapsid protein’s nucleic acid chaperone function and retroviral replication — Supplementary Data 

# Differential contribution of basic residues to HIV-1 nucleocapsid protein’s nucleic acid chaperone function and retroviral replication

## Supplementary Data

files

**Files in this Data Supplement:**

- Supplementary Data - pdf file
