# Supplementary material for: Differential contribution of basic residues to HIV-1 nucleocapsid protein’s nucleic acid chaperone function and retroviral replication
Source: Nucleic Acids Res. 2013 Nov 28;42(4):2525–37. doi: 10.1093/nar/gkt1227 (PMC3936775; doi:10.1093/nar/gkt1227)
Supplement: Supplementary Data [file supp_gkt1227_nar-02998-h-2013-File008.pdf]

Supplementary Data

For the manuscript "Differential contribution of basic residues to HIV-1 nucleocapsid protein's nucleic acid chaperone function and retroviral replication", by Hao Wu, Mithun Mitra, Micah J. McCauley, Robert J. Gorelick, Ioulia Rouzina, Karin Musier-Forsyth and Mark C. Williams

Figure S1	Plots demonstrating the correlation of *in vitro* measurements of RNA or DNA binding affinity or RNA/DNA annealing with replication measurements for wild type and mutant HIV-1 NC in cells.
Figure S2	Plots demonstrating the correlation of *in vitro* single molecule measurements of DNA stretching slope or hysteresis with replication measurements for wild type and mutant HIV-1 NC in cells.

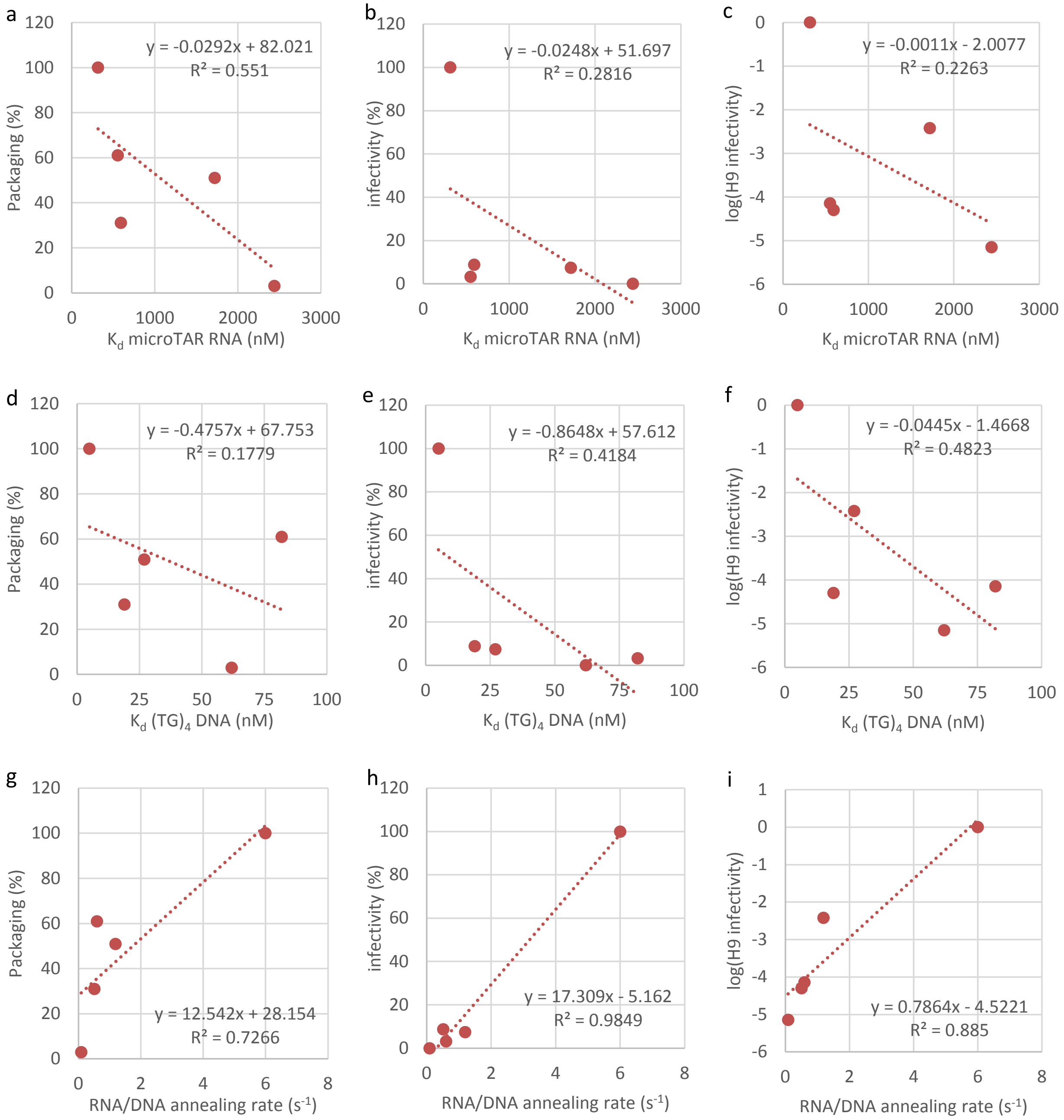

Figure S1. Correlation plots for measurements of in vitro wild type and mutant HIV-1 NC binding to microTAR RNA (a-c), $(TG)_4$ DNA (d-f), and measurements of the TAR RNA/DNA annealing rate (g-i), for comparison to replication measurements (genomic RNA packaging [a,d,g], single round infectivity [b,e,h], and multiple round infectivity [c,f,i]) in the presence of the same proteins. The square of the correlation coefficient and equation for each linear fit is shown in the graph, and the resulting correlation coefficient is presented in Table 3 in the main manuscript.

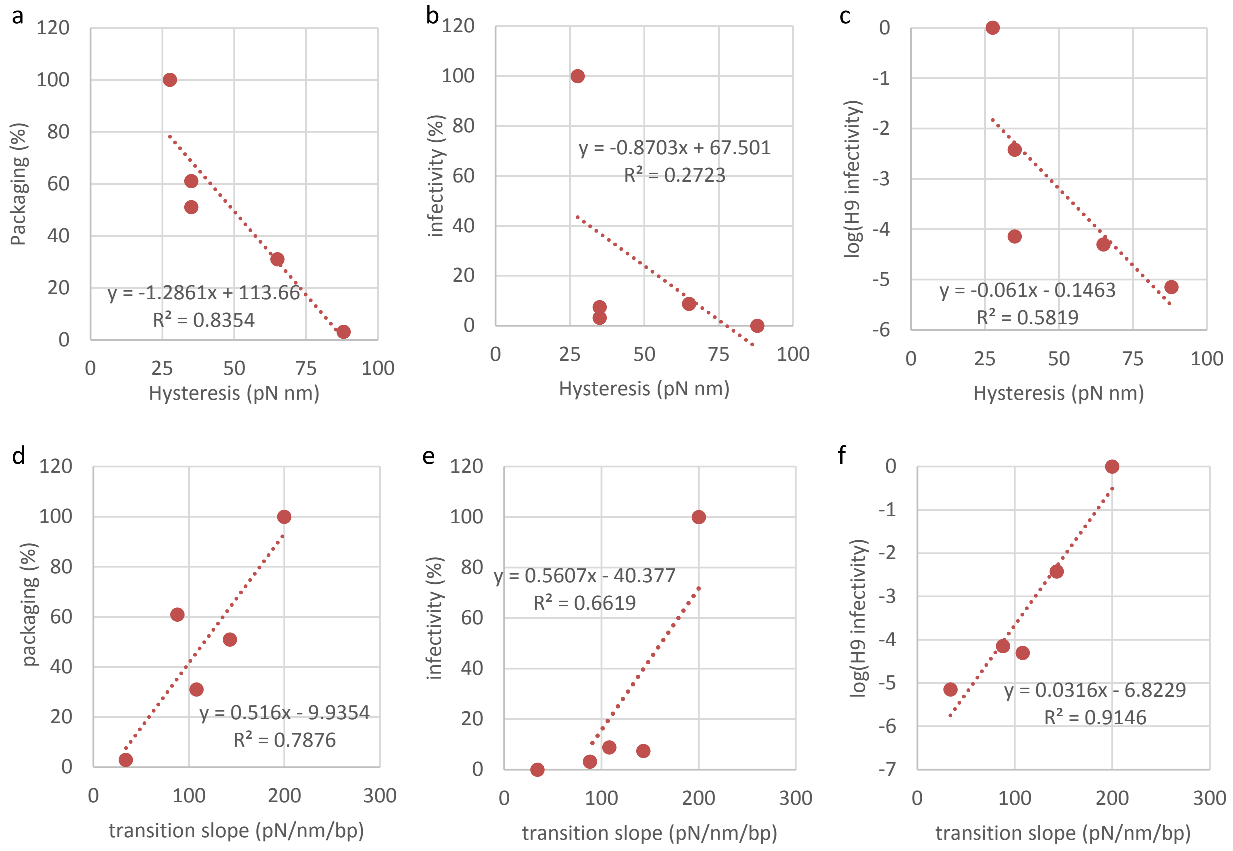

Figure S2. Correlation plots for measurements of in vitro single molecule DNA stretching hysteresis (a-c) and single molecule DNA stretching transition slope (d-f), for comparison to replication measurements (genomic RNA packaging [a,d], single round infectivity [b,e], and multiple round infectivity [c,f]) in the presence of the same proteins. The square of the correlation coefficient and equation for each linear fit is shown in the graph, and the resulting correlation coefficient is presented in Table 3 in the main manuscript.
